# Reactive surveillance of suicides during the COVID-19 pandemic in France, 2020 to March 2022

**DOI:** 10.1017/S2045796023000148

**Published:** 2023-04-17

**Authors:** Anne Fouillet, Diane Martin, Isabelle Pontais, Céline Caserio-Schönemann, Grégoire Rey

**Affiliations:** 1 Santé publique France, Division for Data Science, Saint-Maurice, France; 2 Epidemiological Centre on Medical Causes of Death CépiDc–National Institute of Health and Medical Research (INSERM), Kremlin-Bicêtre, France

**Keywords:** epidemiology, mental health, suicide, validation study, causes of death

## Abstract

**Aims:**

Mitigation actions during the COVID-19 pandemic may impact mental health and suicide in general populations. We aimed to analyse the evolution in suicide deaths from 2020 to March 2022 in France.

**Methods:**

Using free-text medical causes in death certificates, we built an algorithm, which aimed to identify suicide deaths. We measured its retrospective performances by comparing suicide deaths identified using the algorithm with deaths which had either a Tenth revision of the International Classification of Diseases (ICD-10) code for ‘intentional self-harm’ or for ‘external cause of undetermined intent’ as the underlying cause. The number of suicide deaths from January 2020 to March 2022 was then compared with the expected number estimated using a generalized additive model. The difference and the ratio between the observed and expected number of suicide deaths were calculated on the three lockdown periods and for periods between lockdowns and after the third one. The analysis was stratified by age group and gender.

**Results:**

The free-text algorithm demonstrated high performances. From January 2020 to mid-2021, suicide mortality declined during France’s three lockdowns, particularly in men. During the periods between and after the two first lockdowns, suicide mortality remained comparable to the expected values, except for men over 85 years old and in 65–84 year-old age group, where a small number of excess deaths was observed in the weeks following the end of first lockdown, and for men aged 45–64 years old, where the decline continued after the second lockdown ended. After the third lockdown until March 2022, an increase in suicide mortality was observed in 18–24 year-old age group for both genders and in men aged 65–84 years old, while a decrease was observed in the 25–44 year-old age group.

**Conclusions:**

This study highlighted the absence of an increase in suicide mortality during France’s COVID-19 pandemic and a substantial decline during lockdown periods, something already observed in other countries. The increase in suicide mortality observed in 18–24 year-old age group and in men aged 65–84 years old from mid-2021 to March 2022 suggests a prolonged impact of COVID-19 on mental health, also described on self-harm hospitalizations and emergency department’s attendances in France. Further studies are required to explain the factors for this change. Reactive monitoring of suicide mortality needs to be continued since mental health consequences and the increase in suicide mortality may be continued in the future with the international context.

## Introduction

The emergence of SARS CoV-2 internationally in January 2020 and the rapid spread of the virus throughout the population in France led to the implementation of the country’s first lockdown from mid-March to mid-May 2020. This mitigation action resulted in a sudden change in the population’s habits, followed by a progressive return to a somewhat normal life until the end of the summer. In October 2020, the second wave of the pandemic led to new mitigation restrictions, including local and national curfews, a 6-week lockdown and the interruption of all cultural and social activities (Gouvernement Français, 2021). A third 4-week lockdown was also led in April 2021 as a complement to other mitigation and prevention actions, like mandatory face mask in public places or a vaccine campaign from January 2021.

In the context of the COVID-19 pandemic, uncertainty about the future, social isolation due to stay-at-home orders, people’s economic situation, ambivalent statements by political representatives and anxiety-provoking information by the mass media may all lead to higher rates of anxiety, depression and suicides in the general public (Hossain *et al.*, 2020). This is especially true for individuals with pre-existing psychiatric conditions, people with low resilience and individuals who live in regions with areas having high COVID-19 prevalence (Hossain *et al.*, 2020). However, recent studies highlighted that suicide mortality has actually decreased in several countries during the COVID-19 crisis, and more specifically during lockdown periods (John *et al.*, 2020; Pirkis *et al.*, 2022). The association between the health crisis and suicide in France deserves specific attention, as the country has one of the highest suicide rates in Western Europe (Eurostat, 2021) and because socio-cultural factors which influence suicide may be very country dependent.

The search for reactive indicators to monitor and track changes in mental health (depression, anxiety, dark thoughts and suicide ideation) during the ongoing COVID-19 pandemic is an international concern. Several sources have been explored, such as calls to telephone helplines (Zalsman *et al.*, 2021) and internet queries via Google Trends (Ayers *et al.*, 2021; Halford *et al.*, 2020). However, these sources only provide an indirect and partial overview of the suicide mortality situation, as they measure the lack of well-being without any indication of a suicidal act, and without individual demographic information to better characterize the populations involved.

Mortality surveillance in France is based on death certificates filled out by medical practitioners. These certificates are designed according to the international standard recommended by the World Health Organization (WHO) (World Health Organization, 2016). The ‘cause of death’ section comprises of two parts: in part one, the sequence of morbid conditions is reported in descending order from the immediate to the underlying causes of death (4 lines); in part two, unrelated but contributory causes of death are reported (2 lines).

Each part consists of free-text fields where physicians write down one or more causes of death using one or more words. The French Epidemiology Centre on Medical Causes of Death (Inserm-CépiDc) is responsible for the French national causes of death database. It implements the coding process following WHO rules, from the Tenth revision of the International Classification of Diseases (ICD-10) (World Health Organization, 2016). It can take between 4 and 6 months for the free-text medical causes of death (FT-MCD) to be made available to epidemiologists and even longer for the subsequent coding.

Since 2007, an Electronic Death Registration System (EDRS) allows physicians to certify deaths electronically. The added-value of the EDRS for reactive mortality surveillance is that free-text causes of death are sent within a few minutes of death certificate validation and are immediately readable. The drawbacks of this system are that (i) it is not deployed uniformly according to region and place of death and (ii) it over-represents hospital-based deaths (Fouillet *et al.*, 2019).

According to recent work (Baghdadi *et al*., 2019a, 2019b), some Natural Language Processing approaches are relevant for the fully automatic detection of specific nosologic entities, as they have a sufficient level of sensitivity and specificity for reactive and consistent mortality monitoring.

The objective of this study was to evaluate an algorithm to identify death certificates where suicide was mentioned in both the free-text ‘cause of death’ parts and to describe the temporal evolution of suicides from January 2020 to March 2022 during the COVID-19 pandemic in France.

## Methods

We first described the data to be used for this study. We then defined an algorithm to identify suicide deaths and how the algorithm performances would be evaluated. Finally, we analysed the algorithm-based number of suicide deaths during the COVID-19 period and compared this with a baseline estimated number using a regression model over a 5-year historical period.

### Data

All death certificates for the period 2015 to March 2022 in France were available.

For the period 2015 to 2017, for each certificate, medical causes of deaths were coded using ICD-10 codes and an underlying cause of death was identified. FT-MCD written by medical practitioners was also available for all certificates.

For the period 2018 to March 2022, only FT-MCD written by medical practitioners were analysed in the present study, since ICD-10 coding was still ongoing when the study started. Furthermore, the number of deaths on this period is still provisional, as a very limited part of paper death certificates is usually missing (less than 2% of total number of deaths).

The use of mortality data in the frame of public health surveillance has been authorized by the French National Commission for Data protection and Liberties (CNIL).

### Algorithm to identify death certificates with medical causes referring to suicide

Based on FT-MCD, we searched for expressions (clusters of words and expressions) of medical causes of death which indicated suicide. Supplementary terms and expressions indicative of a violent non-suicidal death were identified to exclude these deaths. The expressions were searched for in the two certificates’ sections of cause of death. The final algorithm identifying suicide deaths is available in the Supplementary file.

### Performance measures of the algorithm

In accordance with previous studies, we defined suicide deaths as having an ICD-10 ‘intentional self-harm’ code from X60 to X84.9 as the underlying cause in the death certificate. Furthermore, given that previous work highlighted that deaths with ‘an external cause of undetermined intent’ frequently mask a suicide in France (Aouba *et al*., 2011), we also considered that deaths with ICD-10 codes Y10 to Y34 and Y87.2 as the underlying cause were suicide deaths.

Death certificates where the underlying cause corresponded to any of these ICD-10 codes were used as the gold-standard to measure the performance of the FT-MCD algorithm.

The selected terms and expressions included in the algorithm were initially inspired from the Inserm-CépiDc dictionary, which contains all the different expressions of causes of death found at least once in the free-text areas of death certificates since the early 2000s and the associated recorded (i.e., coded) ICD-10 code. Based on this initial list of expressions, the algorithm was applied to identify suicide mortality data for 2016. The performance of the algorithm in terms of sensitivity and predictive positive value was improved iteratively by adding or removing missing terms or expressions to the algorithm, based on an analysis of non-classified or misclassified death certificates (i.e., identified with an ICD-10 code but not with the algorithm or vice-versa, respectively), compared with the gold-standard.

The final performance analysis was then conducted on data for the 2015 to 2017 period. Sensitivity, predictive positive value and F-measure (harmonic mean of the two previous measures) were calculated for each year for paper and electronic certificates, which were considered separately in order to check that the text mentioned was comparable between the two sources and then together.

The correlation coefficients between the weekly number of deaths with an ICD-10 ‘intentional self-harm’ or ‘external cause of undetermined intent’ code and the weekly number of suicide deaths based on the FT-MCD algorithm were also calculated.

### Evolution of suicide deaths from 2020 to March 2022

The final FT-MCD algorithm was then applied to identify suicide deaths among all death certificates (paper + electronic) from January 2015 to March 2022.

The weekly number of suicide deaths from 2020 to 27 March 2022 was compared with a baseline number, estimated with data from 2015 to 2019 using a generalized additive model (GAM), which included a linear annual trend, a week effect using a spline and an interaction effect between age group (11–17, 18–24, 25–44, 45–64, 65–84 and more than 85 years old) and gender. Age groups were chosen in accordance with those used for morbidity surveillance in mental health set up in 2020 (Santé Publique France, 2022).

We focused on France’s three national lockdown periods in 2020 (Week 11–Week 19 and W44–W51) and 2021 (W14–W17), on the period between these lockdowns (W20–W43, 2020 and W52, 2020–W13, 2021) and a period after the third lockdown ended (W18, 2021–W12, 2022). The difference and the ratio between the observed number of suicide deaths and the baseline number were calculated by age group and gender. Confidence intervals of the ratios were calculated, considering a negative Binomial distribution.

## Results

### Performance measures of the algorithm – 2015–2017

**Fig. 1.**
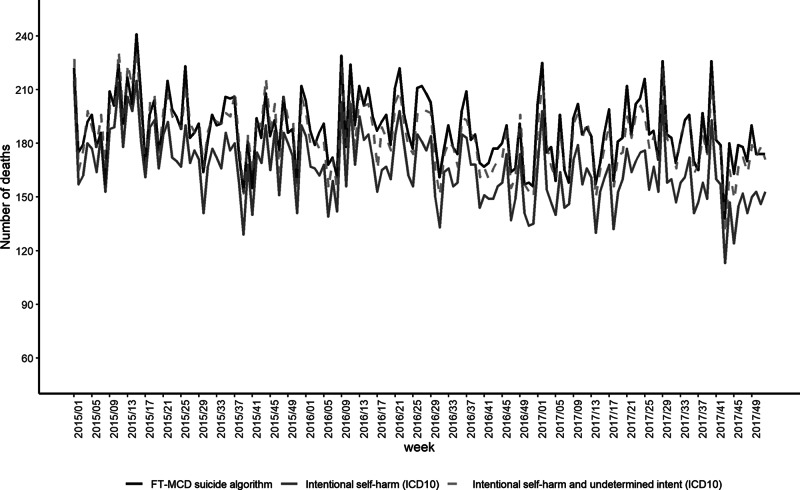
Weekly number of (i) suicide deaths coded with ‘intentional self-harm’ as underlying cause, (ii) deaths from suicide coded with ‘intentional self-harm’ or ‘external cause of undetermined intent’ as underlying cause and (iii) suicide deaths based on FT-MCD, from 2015 to 2017, France.

The weekly variations in the number of FT-MCD suicide deaths from 2015 to 2017 were correlated to the weekly variations in the number of deaths with an ICD-10 ‘intentional self-harm’ code as the underlying cause of death (correlation coefficient = 0.93) (Fig. 1). This correlation was stronger when considering the weekly variations in the number of deaths coded as ‘intentional self-harm’ or ‘external cause of undetermined intent’ (correlation coefficient = 0.97). The observed number of suicide deaths (i.e., with the algorithm) was very close to the ICD-10-recorded (i.e., gold-standard) number of deaths when considering ‘intentional self-harm’ and ‘external cause of undetermined intent’ combined (Fig. 1).

Considering all death certificates (electronic + paper) from 2015 to 2017, the algorithm’s sensitivity compared with deaths with ‘intentional self-harm’ as an underlying cause of death was 0.96, while the predictive positive value was equal to 0.85 (Table 1). The sensitivity slightly decreased when we considered deaths coded as either ‘intentional self-harm’ or ‘an external cause of undetermined intent’ (0.93). However, the predictive positive value improved, reaching 0.91. Performance results were similar according to year.

**Table 1.** Performance measures of the FT-MCD algorithm identifying suicide deaths, compared with deaths with an ICD-10 ‘intentional self-harm’ or ‘external cause of undetermined intent’ code as underlying cause of death, from 2015 to 2017, for electronic and paper death certificates

	Intentional self-harm				Either intentional self-harm or external cause of undetermined intent			
	2015	2016	2017	2015−2017	2015	2016	2017	2015−2017
E-death certificates								
Sensitivity	0.96	0.97	0.95	0.96	0.91	0.93	0.89	0.91
Predictive positive value	0.79	0.76	0.75	0.77	0.86	0.83	0.84	0.84
F-measure	0.87	0.85	0.84	0.85	0.88	0.88	0.86	0.87
Paper death certificates								
Sensitivity	0.95	0.96	0.97	0.96	0.92	0.95	0.93	0.93
Predictive positive value	0.87	0.86	0.83	0.85	0.92	0.92	0.91	0.92
F-measure	0.91	0.91	0.89	0.90	0.92	0.93	0.92	0.92
All death certificates								
Sensitivity	0.95	0.96	0.97	0.96	0.92	0.95	0.93	0.93
Predictive positive value	0.87	0.85	0.83	0.85	0.92	0.91	0.91	0.91
F-measure	0.91	0.90	0.89	0.90	0.92	0.93	0.92	0.92

Performance was slightly better when considering paper death certificates (F-measure = 0.92) instead of electronic death certificates (F-measure = 0.87) (Table 1).

### Evolution of suicide deaths from January 2020 to March 2022

From 1 January 2020 to 27 March 2021, 20,175 suicide deaths were counted in France (9089 deaths in 2020, 9062 deaths in 2021 and 2024 deaths from January to 27 March 2022) (Table 2). The number of deaths in 2020 was slightly lower than the average number of deaths during the 5-year reference period (mean number = 9736). Men represented 75.6% of these deaths. The highest number of deaths was observed in people aged 45–64 years old (36.9%), while 6.0% were aged less than 24 years old, 21.9% were aged 25–44 years old and 25.8% were aged 65–84 years old. The distribution of suicide deaths by age over the study period was significantly different to that recorded during the reference period but that by gender was similar between the two periods (Table 2).

**Table 2.** Distribution of suicide deaths from W01-2020 to W12-2022 and from 2015 to 2019 in France

Suicide deaths from 2020 to 2022 (March)			Suicide deaths over the reference period from 2015 to 2019			Chi^2^ test
	*N*	%		*N*	%	*p*-value
Total	20,175	–	Total	48,678	–	
2020	9089		2015	9997		
2021	9062		2016	9792		
2022 (Jan–March)	2024		2017	9578		
			2018	9898		
			2019	9413		
			2015−2019 (mean)	9736		
Men	15,246	75.57	Men	36,708	75.41	0.25
Women	4924	24.41	Women	11,944	24.54	
Missing	5	0.02	Missing	26	0.05	
00−10 years old	49	0.24	00−10 years old	136	0.28	<0.005
11−17 years old	246	1.22	11−17 years old	548	1.13	
18−24 years old	908	4.50	18−24 years old	1826	3.75	
25−44 years old	4418	21.90	25−44 years old	11,265	23.14	
45−64 years old	7436	36.86	45−64 years old	18,828	38.68	
65−84 years old	5195	25.75	65−84 years old	11,789	24.22	
≥85 years	1923	9.53	≥85 years	4282	8.80	
Missing	0		Missing	4	0.01	
Home	12,741	63.15	Home	30,017	61.66	<0.005
Public hospital	2576	12.77	Public hospital	5882	12.08	
Private clinic	269	1.33	Private clinic	390	0.80	
Nursing home	407	2.02	Nursing home	838	1.72	
Public place	2263	11.22	Public place	5226	10.74	
Other or missing	1919	9.51	Other or missing	6325	12.99	

From January 2020 to March 2022, the majority of suicide deaths occurred at home (63.2% vs. 61.7% over the reference period), while 12.8% took place in public hospitals (vs. 12.1% over the reference period) and 11.2% in public places (vs. 10.7%) (Table 2).

**Fig. 2.**
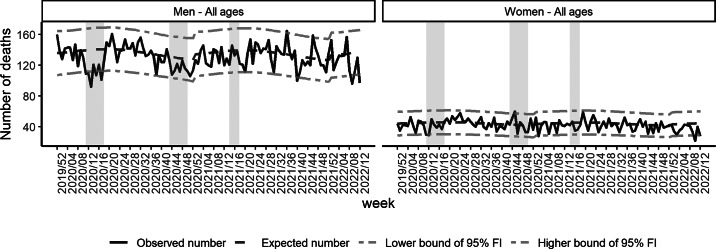
Observed and expected numbers of suicide deaths and 95% fluctuation interval by gender, from W01-2020 to W12-2022, France.

The weekly number of observed suicide deaths was similar to the baseline number before the first lockdown in all age groups and both genders. A marked decrease compared with the baseline was observed from W12 to W19-2020 (first lockdown) for all ages and both genders (ratio = 0.81 [0.76–0.86]). This decrease was higher in men than in women and was higher in the 45–64 year-old age group (Fig. 2 and Table 3).

**Table 3.** Observed (O) number of suicide deaths, excess deaths (O–E) and mortality ratio (O/E) by age group and gender, during the different periods studied from March 2020 to March 2022 (Week 12), France

		Men				Women				Both genders			
	Age	O	O–E	O/E		O	O–E	O/E		O	O–E	O/E	
First lockdown (W12–W19)	11−17	9	−1	0.87	[0.43−1.76]	2	−4	0.31	[0.08−1.31]	11	−6	0.66	[0.35−1.23]
	18−24	37	−6	0.85	[0.60−1.21]	15	3	1.21	[0.69−2.12]	52	−4	0.93	[0.69−1.25]
	25−44	*226*	*−49*	*0.82*	*[0.71−0.94]*	60	−9	0.87	[0.66−1.14]	*286*	*−59*	*0.83*	*[0.73−0.94]*
	45−64	*314*	*−117*	*0.73*	*[0.65−0.82]*	*112*	*−33*	*0.77*	*[0.63−0.95]*	*426*	*−150*	*0.74*	*[0.67−0.82]*
	65−84	*211*	*−55*	*0.79*	*[0.68−0.92]*	92	−2	0.98	[0.78−1.22]	*303*	*−58*	*0.84*	*[0.74−0.95]*
	85 and over	88	−6	0.94	[0.75−1.18]	36	−1	0.96	[0.67−1.38]	124	−7	0.95	[0.78−1.15]
	All ages	*888*	*−235*	*0.79*	*[0.74−0.85]*	*318*	*−48*	*0.87*	*[0.77−0.98]*	*1206*	*−283*	*0.81*	*[0.76−0.86]*
Inter-period (W20–W43)	11−17	30	0	0.99	[0.67−1.46]	*8*	*−11*	*0.43*	*[0.21−0.88]*	38	−11	0.77	[0.55−1.09]
	18−24	138	10	1.08	[0.90−1.30]	48	11	1.31	[0.96−1.81]	186	22	1.13	[0.97−1.33]
	25−44	754	−55	0.93	[0.86−1.01]	189	−14	0.93	[0.80−1.09]	943	−68	0.93	[0.87−1.00]
	45−64	1252	−14	0.99	[0.93−1.05]	431	7	1.02	[0.92−1.13]	1683	−7	1.00	[0.95−1.05]
	65−84	838	56	1.07	[0.99−1.15]	*319*	*42*	*1.15*	*[1.02−1.30]*	*1157*	*98*	*1.09*	*[1.03−1.16]*
	85 and over	*319*	*44*	*1.16*	*[1.03−1.31]*	98	−12	0.89	[0.72−1.11]	417	33	1.08	[0.98−1.21]
	All ages	3339	42	1.01	[0.98−1.05]	1096	23	1.02	[0.96−1.09]	4435	65	1.01	[0.98−1.05]
Second lockdown (W44–W51)	11−17	14	4	1.46	[0.81−2.66]	9	3	1.53	[0.73−3.23]	23	8	1.49	[0.94−2.37]
	18−24	41	1	1.02	[0.73−1.43]	17	6	1.48	[0.86−2.54]	58	6	1.12	[0.85−1.49]
	25−44	221	−33	0.87	[0.75−1.00]	56	−8	0.88	[0.66−1.17]	*277*	*−41*	*0.87*	*[0.77−0.99]*
	45−64	*332*	*−66*	*0.83*	*[0.74−0.94]*	144	11	1.08	[0.90−1.29]	*476*	*−56*	*0.90*	*[0.81−0.99]*
	65−84	225	−21	0.91	[0.79−1.05]	*65*	*−22*	*0.75*	*[0.58−0.97]*	*290*	*−43*	*0.87*	*[0.77−0.99]*
	85 and over	102	16	1.18	[0.95−1.46]	47	13	1.36	[0.99−1.88]	*149*	*28*	*1.23*	*[1.03−1.47]*
	All ages	*935*	*−102*	*0.90*	*[0.84−0.97]*	340	3	1.01	[0.90−1.13]	*1275*	*−99*	*0.93*	*[0.87−0.98]*
Inter-lockdown (W52/2020—W13/2021)	11−17	19	0	1.02	[0.62−1.66]	13	1	1.13	[0.62−2.06]	32	2	1.06	[0.72−1.55]
	18−24	80	1	1.02	[0.80−1.29]	26	4	1.16	[0.75−1.77]	106	5	1.05	[0.85−1.29]
	25−44	481	−17	0.97	[0.88−1.07]	121	−4	0.97	[0.80−1.18]	602	−20	0.97	[0.89−1.06]
	45−64	*674*	*−105*	*0.86*	*[0.80−0.94]*	227	−34	0.87	[0.75−1.00]	*901*	*−140*	*0.87*	*[0.81−0.93]*
	65−84	474	−7	0.98	[0.89−1.09]	180	10	1.06	[0.90−1.24]	654	2	1.00	[0.92−1.09]
	85 and over	175	6	1.03	[0.88−1.22]	60	−7	0.89	[0.68−1.17]	235	−2	0.99	[0.86−1.14]
	All ages	*1911*	*−119*	*0.94*	*[0.90−0.99]*	628	−32	0.95	[0.87−1.04]	*2539*	*−151*	*0.94*	*[0.90−0.98]*
Third lockdown (W14—W17/2021)	11−17	5	0	0.97	[0.37−2.54]	3	0	0.95	[0.28−3.26]	8	0	0.97	[0.45−2.06]
	18−24	24	2	1.11	[0.72−1.73]	9	3	1.46	[0.69−3.07]	33	5	1.19	[0.81−1.74]
	25−44	133	−4	0.97	[0.81−1.17]	30	−4	0.88	[0.60−1.29]	163	−8	0.95	[0.81−1.13]
	45−64	*176*	*−38*	*0.82*	*[0.70−0.97]*	55	−17	0.77	[0.58−1.02]	*231*	*−54*	*0.81*	*[0.70−0.93]*
	65−84	146	14	1.11	[0.92−1.32]	42	−5	0.90	[0.65−1.25]	188	9	1.05	[0.90−1.23]
	85 and over	54	8	1.16	[0.87−1.56]	21	2	1.13	[0.71−1.82]	75	10	1.16	[0.90−1.49]
	All ages	538	−19	0.97	[0.88−1.06]	160	−21	0.88	[0.75−1.05]	698	−40	0.95	[0.87−1.03]
Post-lockdown (W18/2021—W12/2022)	11−17	66	8	1.13	[0.87−1.48]	45	9	1.26	[0.91−1.74]	111	17	1.18	[0.96−1.45]
	18−24	*287*	*42*	*1.17*	*[1.03−1.33]*	*88*	*18*	*1.26*	*[1.00−1.59]*	*375*	*60*	*1.19*	*[1.07−1.33]*
	25−44	*1379*	*−171*	*0.89*	*[0.84−0.94]*	*323*	*−65*	*0.83*	*[0.74−0.94]*	*1702*	*−236*	*0.88*	*[0.83−0.92]*
	45−64	*2222*	*−204*	*0.92*	*[0.88−0.96]*	*732*	*−81*	*0.90*	*[0.83−0.97]*	*2954*	*−285*	*0.91*	*[0.88−0.95]*
	65−84	*1594*	*95*	*1.06*	*[1.01−1.12]*	513	−17	0.97	[0.88−1.06]	2107	78	1.04	[0.99−1.09]
	85 and over	552	25	1.05	[0.96−1.15]	209	−1	0.99	[0.86−1.15]	761	24	1.03	[0.96−1.12]
	All ages	*6115*	*−204*	*0.97*	*[0.94−0.99]*	*1916*	*−140*	*0.93*	*[0.89−0.98]*	*8031*	*−343*	*0.96*	*[0.94−0.98]*

Values in italics indicate significant mortality ratio.

Between the country’s first two lockdowns (W20–W43, 2020), the number of suicide deaths was comparable to the baseline, except for men aged 85 years old and over and both genders aged 65–84 years old (Table 3). For these two groups, the number of deaths was significantly higher than the baseline (ratio = 1.16 [1.03–1.31] and ratio = 1.09 [1.03–1.16], respectively).

**Fig. 3.**
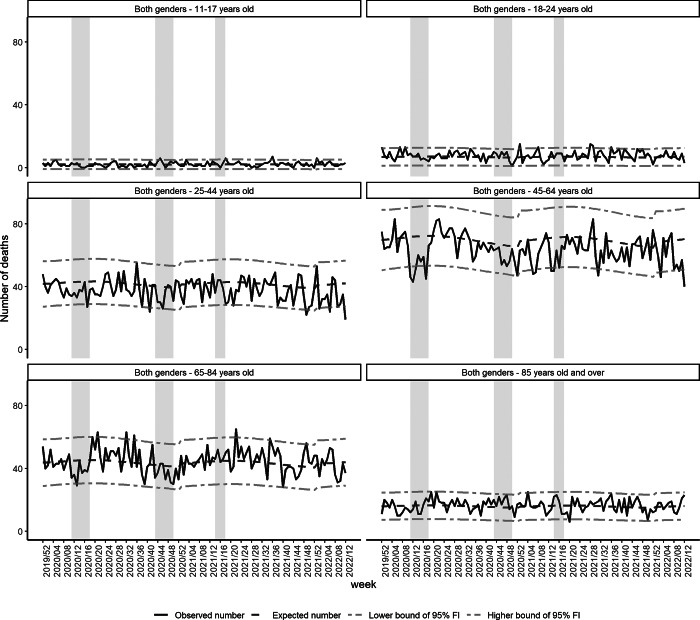
Observed and expected numbers of suicide deaths and 95% fluctuation interval by age group, from W01-2020 to W12-2022, France.

During the second lockdown (W44–W51, 2020), the observed number of deaths was also significantly lower than the baseline (ratio for all ages and gender = 0.93 [0.87–0.98]). More specifically, this decrease was observed in men aged 45–64 years old and women aged 65–84 years old (Fig. 3 and Table 3).

The decrease in men aged 45–64 years old continued after the country’s second lockdown, during the third lockdown and after the country’s third lockdown. In men of other age groups and in women of all age groups, the number of deaths was similar to the baseline after the second lockdown and during the third lockdown.

After the country’s third lockdown until W12, 2022, the number of suicide deaths was significantly higher than the baseline for men aged 65–84 years old (ratio = 1.06 [1.01-1.12]) and in the 18–24 year-old age group for both genders (ratio = 1.19 [1.07–1.33]), while a decrease was observed in the 25–44 year-old age group.

## Discussion

Based on FT-MCD certificates, this nationwide study analysed the pattern of suicide deaths by age and gender and its temporal evolution during the COVID-19 pandemic in France from January 2020 to March 2022.

No increase in suicide deaths in France was found for the period March 2020 to April 2021 (end of the third lockdown). Conversely, a decline in suicide deaths was observed during the three lockdown periods (sharper during the first lockdown) particularly in men. During the periods between and after the two first lockdowns, suicide mortality remained comparable to expected values, except for men over 85 years old and in 65–84 year-old age group, where a small number of excess deaths was observed in the weeks following the end of first lockdown and for men aged 45–64 years old where the decline continued after the second lockdown ended. After the third lockdown until March 2022 (W12), an excess death was observed in the 18–24 year-old age group for both genders while a decrease was observed in the 25–44 year-old age group. A small number of excess deaths was also observed for men aged 65–84 years old.

Changes in suicide mortality during the COVID-19 pandemic vary between countries. While remaining unchanged in some nations, decreases were observed from March to July 2020 in 12 out of 21 countries as well as in China and India (Behera *et al.*, 2021; Pirkis *et al.*, 2021; Zheng *et al.*, 2021). This decline could be related to the implementation of strict public health protection measures (stay-at-home orders, school closures, reduction of business activities, etc.) and to a sense of collectivity when a major crisis occurs affecting the whole population (Carr *et al.*, 2021; Eguchi *et al.*, 2021; Radeloff *et al.*, 2021). Furthermore, Japan saw an excess suicide mortality in women (all age groups) and in men aged 20–29 and over 80 years old from July to November 2020 (Eguchi *et al.*, 2021) (after the first lockdown). Beyond Japan in 2020, the study conducted by Pirkis *et al.* on data from 33 countries or areas-within-countries during the first 9 to 15 months of the pandemic showed no consistent patterns across age group and gender, except for Japan and New Delhi where a greater-than-expected number of suicides were noticed in all or almost analysis by age and gender and inversely for Brazil and England and Wales (Pirkis *et al.*, 2022). Those results were also observed in Germany (Radeloff *et al.*, 2022).

The present study found an overall decline in the number of suicide deaths in France mainly during the first lockdown, followed by a return to the usual pattern, except for people aged 65–84 years old and men over 85 years old. However, the scope of the study did not allow us to conclude whether other mental health events with less acute consequences were impacted or not by the pandemic. Both of France’s nationwide lockdowns in 2020 and 2021 may have generated contradictory effects depending on the population. More specifically, while adverse psychosocial effects have been widely observed in young people and children (Monnier *et al.*, 2021; Wathelet *et al.*, 2020), possible positive effects were also seen in adults, thanks to a reduction in everyday stress or in stress due to normal social relationships for people with mental health disorders. The increase in suicide mortality observed in the 18–24-year-old and 65–84-years old age groups after the third lockdown until March 2022 could reflect a prolonged impact of the epidemic, also described on self-harm hospitalizations in France (Jollant *et al.*, 2022) and in emergency department’s attendances for mental health (Santé Publique France, 2022).

Further, a higher risk of suicide is observed for people with mental illness and studies have shown an impact of the pandemic on the clinical course of mental illness like bipolar disorders (Fornaro *et al.*, 2021). The individual information declared on death certificates about the mental history and healthcare of the patient prior to death are too limited to be able to provide robust analysis of the characteristics of patients who died. In particular, a physician who declares a suicide is not systematically the referring physician of the deceased person and does not know his possible psychiatric conditions. Complementary studies using data from the National Hospitalization Database (PMSI) and about the reimbursements of medical care could be conducted in order to better explore the health status of suicidants during the pandemic in comparison with a prior reference period, following the design of a previous study (Laanani *et al.*, 2020).

Beyond the indirect impacts of the COVID-19 epidemic, the mental health consequences and the increase in suicide mortality may be continued in the future, with the international context related to the war in Ukraine. Reactive monitoring of suicide mortality therefore needs to be continued.

### Limitations

In the absence of reactive coding of medical causes of deaths, this study generated an algorithm based on free-text medical causes included in death certificates. Although it cannot provide as exact and standardized a quantification of the number of suicide deaths as that obtained when computing the underlying cause of deaths, it nonetheless constitutes a good proxy indicator for routine monitoring of the temporal dynamic of suicide mortality in the context of ensuring reactive surveillance. This approach to analyse FT-MCD can be replicated in many countries and can improve reactive surveillance of suicide or any indicators in other domains. However, that needs to also replicate the language processing analysis to consider possible variations of vocabulary and syntax between countries and periods. One possible barrier to the generalization of the proposed method is the availability of nosologists and medical language experts to develop and maintain these algorithms.

Electronic death certificates are the only data sources which ensure rapid (24–48 hours) mortality surveillance in terms of medical causes of death. The proportion of electronic death certificates among all certificates increased during the pandemic, from 20% in March 2020 to around 34% in March 2022. As showed in results, the performance of the algorithm was close for both electronic and paper certificates. It is highly unlikely that the increase in the part of electronic death certificates in all deaths affect the trends in suicide mortality observed in the present study. Especially, EDRS collects less than 5% of deaths occurring at home from 2020 to 2022, but over 60% of suicides usually occur at home.

Nevertheless, wider deployment of electronic death certification, particularly in nursing homes and in private homes, is required to ensure reactive surveillance of suicide mortality. A better overview of suicide mortality is possible with a 4- to 6-month delay when paper death certificates are available.

## Conclusion

This nationwide study showed that in France, it is possible to monitor suicide mortality reactively based on death certificates with a 4-month delay. This delay will be reduced to a couple of days as electronic death certification is rolled out to physicians, allowing for much more reactive monitoring of a larger panel of diseases.

This study highlighted the absence of an increase in suicide mortality during the COVID-19 pandemic until mid-2021 in France and a substantial decline during the country’s three lockdown periods, reflecting findings in other countries. Noticeably, an increase in suicide mortality was observed in 18–24-year-old and 65–84-year-old age groups from mid-2021 to March 2022 that could reflect a prolonged impact of COVID-19 on mental health. Further studies are required to explain this change.

## Data Availability

The complete dataset of this research is only available under strictly secured conditions as it contains individual data protected by French legislation. Aggregated data could be shared on reasonable request to the corresponding author.

## Supplementary material

For supplementary material accompanying this paper visit http://doi.org/10.1017/S2045796023000148. click here to view supplementary material
