# Supplementary material for: Reactive surveillance of suicides during the COVID-19 pandemic in France, 2020 to March 2022
Source: Epidemiol Psychiatr Sci. 2023 Apr 17;32:e20. doi: 10.1017/S2045796023000148 (PMC10130823; doi:10.1017/S2045796023000148)
Supplement: Supplementary file 1 [file S2045796023000148sup001.docx]

**Reactive surveillance of suicides during the COVID-19 epidemic in France, 2020- 2021**

**European Journal of Epidemiology**

**Anne Fouillet^1^, Diane Martin^2^, Isabelle Pontais^1^, Céline Caserio-Schönemann^1^, Grégoire Rey^2^**

**Corresponding author:** Anne Fouillet

Santé publique France, Division for Data Science, Saint-Maurice, France

[Anne.fouillet@santepubliquefrance.fr](mailto:Anne.fouillet@santepubliquefrance.fr)

**Box S1:** List of expressions and terms used to identify suicide deaths in free-text medical causes of death

**Search terms:**

PENDAIS* OR PENDU OR PNEDAIS* OR PENDASI* OR PENDIAS* OR PENSAIS*

OR STRANGUL* OR ASPHYXIE MECAN* OR (SAC AND POUBE*) OR (SAC AND PLAST) OR (PERCU* AND TRAIN) OR (PRECU* AND TRAIN) OR DEFENEST* OR AUTOINTOX* OR AUTO-INTOX* OR (INTOXIC* AND MED*)OR TS MEDIC* OR TA MEDIC* OR AUTO INTOX* OR IMV OR IVM OR BALISTI* OR (ARME AND FEU)OR (COUP AND FUSIL)OR ARME BLANC OR GRANDE HAUTEUR OR (CHUTE AND ETAG*) OR (CHUTE AND METR* AND ^ENDOMETR)OR IMMOLATION OR VOLONTAI* OR AUTOLYSE OR AUTOYSE OR AUTOLYTI* OR AUTOINFLIG* OR AUTO INFLIG* OR AUTO-INFLIG* OR (INGESTION AND CAUSTIQUE)OR (INJESTION AND CAUSTIQUE) OR (INHALATION AND CAUSTIQUE)OR (INGESTION AND ACIDE) OR (INJESTION AND ACIDE) OR (INHALATION AND ACIDE) OR (INGESTION AND MEDI) OR (INJESTION AND MEDI*) OR (ABSORB* AND MEDI*) OR (SURDOS* AND MEDI*) OR SUICID OR SCUICID OR DUICID OR SUCID OR TDS OR TS MED OR (TS AND (^ANTECEDENTS OR ^TRAITEMENTS) OR T S OR TA

**Exclusion :**

INVOLONTAI* OR ACCIDENT OR ACCIDETN OR HOMICID* OR MEURTR* OR AGRESSION OR NON SUICID* OR CRIMI* OR (AUTOLY* AND DEMANDE) OR(AUTOLY* AND PREVUE)OR DEFENESTRATION SUR INCENDIE)OR NON VOLONTAI* OR (ARRET AND VOLONTAI*)
